# CBT therapists’ attitudes toward virtual reality use in psychotherapy: a brief report from the Czech Republic and Slovakia

**DOI:** 10.3389/fpsyg.2026.1811278

**Published:** 2026-05-07

**Authors:** Kristína Kvapil Varšová, Vojtěch Juřík

**Affiliations:** Department of Psychology, Masaryk University, Brno, Czechia

**Keywords:** cognitive-behavioral therapy, implementation barriers, therapist attitudes, virtual reality, virtual reality exposure therapy

## Abstract

Virtual reality (VR) is an increasingly accessible technology with strong potential to support cognitive-behavioral therapy (CBT), particularly through virtual reality exposure therapy (VRET). However, real-world uptake remains limited, and there is a lack of knowledge about implementation needs in specific regions. This convergent mixed-methods study explored the attitudes, perceived benefits and barriers, and desired functional and training requirements of Czech and Slovak CBT psychotherapists regarding the use of VR. Thirty-three therapists completed an anonymous online questionnaire combining Likert-type ratings and open-ended questions. Overall attitudes toward VR were positive (M = 4.09, SD = 0.72), while perceived acceptance among colleagues was moderate (M = 3.39, SD = 0.80). Perceived risk of detracting from the therapeutic alliance was low-to-moderate (M = 2.09, SD = 0.84) and differed by practice length, with the lowest risk in mid-career therapists. Key perceived benefits were client safety and controllability of exposure; the dominant barrier was financial cost (60.6%), followed by training and technical concerns. Therapists prioritized safety controls (e.g., stop button), flexible exposure adjustment, biofeedback, and structured protocols, with a strong preference for online training with practical demonstrations. Findings suggest readiness to adopt VR, contingent on affordable infrastructure, standardized guidance, and clinician-centered tool design.

## 1 Introduction

VR is a technology that creates immersive digital environments, allowing users to engage and interact in ways that feel real (Juřík et al., 2023; Moro et al., 2017; Slater, 2018). The sense of presence is a key dimension of VR that intensifies the user’s experience of being in another place through immersive multisensory feedback (Kelly et al., 2023; Steuer, 1992). Owing to its immersive nature, VR enables psychologists to create controlled environments in which clients can confront fears, process emotions, and develop coping strategies, leading to substantial therapeutic benefits (Boeldt et al., 2019).

The strongest application of VR has been in CBT, where it serves as a tool for enhancing the effectiveness of treatments targeting psychological disorders, particularly anxiety and related conditions (Nakao et al., 2021; Opriş et al., 2012; Varšová et al., 2024). One of the primary applications of VR in this context is VRET, which integrates traditional CBT exposure techniques with immersive virtual environments (Kvapil Varšová and Juřík, 2024; Chard and van Zalk, 2022; Ju, 2024). According to Scheveneels et al. (2025), VRET operates through several potential mechanisms of action. These include habituation (the gradual reduction of anxiety through repeated exposure), enhancement of self-efficacy (strengthening the client’s confidence in managing feared situations), and expectancy violation (demonstrating that the anticipated negative outcome does not occur).

VRET has proven to be an effective tool for treating specific phobias, including acrophobia (Chou et al., 2021). Environments used for VRET enable safe and realistic simulations of height-related situations, such as standing on a balcony or climbing tall buildings, which could otherwise be logistically challenging (Arroll et al., 2017). Previous research has shown that even a few VRET sessions can significantly reduce anxiety associated with heights, with long-term effects (Wechsler et al., 2019).

Although research in the field of VR in psychotherapy is advancing and the effectiveness of VRET is increasingly well-documented, its implementation in clinical practice remains limited. Several studies indicate that the attitudes of psychotherapists themselves play a decisive role in the adoption of VR (Jeon et al., 2025; Rimer et al., 2021).

Wray et al. (2023) examined the reasons behind the limited use of VR technology in treating anxiety disorders. The most frequently cited barriers include a lack of evidence from routine clinical practice, specifically, a limited number of comparative studies evaluating the effectiveness of VRET relative to established exposure-based interventions. Importantly, existing research suggests that VRET is often not consistently superior to active control conditions, such as traditional *in vivo* exposure therapy, despite demonstrating comparable efficacy. This highlights a critical gap between technological potential and clinical added value. Additional barriers involve limited therapist awareness, insufficient training in using VR systems, negative perceptions of exposure therapy itself, low patient demand, and institutional caution toward adopting new technologies. Financial and regulatory constraints further complicate adoption, as they often prevent the reimbursement of VR-related costs such as hardware, software licensing, and therapeutic procedures.

Bin et al. (2025) highlight that therapists’ attitudes toward VRET are strongly influenced by the design of the tools they use. Practitioners tend to favor systems that are easy to operate, flexible, provide control over exposure parameters, and align with established therapeutic logic. Their feedback is therefore a crucial input for developing clinically acceptable VR applications.

Rimer et al. (2021) provided important insights into clinicians’ attitudes toward VRET for fear of heights. After directly experiencing VRET, therapists showed a significant increase in confidence in the technology and willingness to use it in practice. The study found that VR scenarios induced discomfort comparable to real-life height situations, and repeated exposure reduced this discomfort. Therapists with prior experience or positive attitudes toward exposure therapy and technology were more open to using VR, and their attitudes improved further after firsthand experience. The authors concluded that VRET can effectively simulate and reduce fear of heights while also fostering more positive attitudes among professionals, thus supporting its broader clinical adoption.

Jeon et al. (2025) identified key factors influencing therapists’ willingness to adopt VRET, including perceived usefulness and social influence, while work-related stress and resistance to change had significant negative effects. These findings demonstrate a strong link between awareness, experience, and readiness to innovate.

To fully utilize VR’s potential in clinical settings, therapeutic teams require not only technical infrastructure but also opportunities for safe hands-on experience, knowledge sharing, and methodological support. Many researchers emphasize the need for training programs, standardized guidelines, and longitudinal studies to track changes in attitudes (Rimer et al., 2021). Investment in infrastructure and legal frameworks is also necessary to support the sustainable integration of VR into psychotherapeutic practice (Bin et al., 2025).

Adoption in clinical practice is a particularly relevant issue in the case of VR because its strongest and most consistent support has been reported for exposure-based treatment of specific phobias, where real-life exposure may be difficult to arrange, costly, or insufficiently controllable. Reviews have shown positive outcomes of VRET across most phobias, particularly acrophobia, and suggest that VR-assisted exposure can achieve effects comparable to *in vivo* exposure (Albakri et al., 2022; Carl et al., 2019; Wechsler et al., 2019). Yet, despite these promising findings, implementation in routine care remains limited, underscoring the importance of examining the factors that may facilitate or hinder clinician uptake (Wray et al., 2023). In this context, the present study contributes to an important but underexplored aspect of the field by examining how specific design features of VR systems, such as usability, controllability, and ecological validity, may influence both therapist acceptance and treatment outcomes. Existing research on therapists’ implementation needs has been conducted predominantly in specific regions, with relatively little attention given to the Central European context. Our study addresses this gap in context-sensitive knowledge by focusing on the Czech and Slovak setting, where empirical evidence on therapists’ implementation needs remains scarce, and by identifying perceived barriers, readiness factors, desired functional features, and preferred training formats that may inform clinician-centered VR tool design and support feasible strategies for introducing and scaling VR-assisted interventions in clinical practice.

## 2 Methods

For this study, a convergent mixed-methods research design was applied. A completely anonymous online questionnaire was used to examine expert psychotherapists’ attitudes and needs related to VR technology and its application in their practice. The questionnaire combined both quantitative and qualitative components. The aim was to reach a broad spectrum of CBT therapists in the Czech Republic and Slovakia while maintaining full respondent anonymity during data collection concerning their attitudes, experiences, and needs related to the use of VR in the therapeutic process. Participation was entirely voluntary and was not financially or otherwise incentivized. Likewise, individuals who chose not to participate or did not complete the questionnaire were not subjected to any pressure to do so.

### 2.1 Participants

Participants were recruited through targeted sharing of the survey on social media, professional networks, and psychotherapy groups in the Czech Republic and Slovakia. Potential respondents were informed that the study focused on psychotherapists’ attitudes and needs related to the use of VR in psychotherapy. The questionnaire was made available online. A total of 33 CBT psychotherapists from the Czech Republic and Slovakia completed the survey. The sample was predominantly female (24 women, 72.7%; 9 men, 27.3%). The majority were early-career therapists, with 60.6% (*n* = 20) having less than 5 years of practice, 30.3% (*n* = 10) with 5–10 years, and only 9.1% (*n* = 3) with over 10 years of clinical experience. Years of clinical practice were grouped (<5, 5–10, >10 years) to reflect typical CBT career stages: training/early practice, post-training consolidation, and established senior practice. Only 5 therapists (15.2%) reported any personal experience using VR in therapy, whereas 28 (84.8%) had never used VR in their practice.

### 2.2 Ethics

The study was reviewed by the Ethics Board of the Department of Psychology, Faculty of Arts, Masaryk University (Brno) and was deemed ethically unproblematic. Because the project involved a fully anonymous online survey, it did not require formal ethics committee review under the university’s procedures and therefore no approval number was issued (Ethics approval exemption guidance is available here). The study was conducted in accordance with applicable guidelines and regulations and adhered to the principles of the Declaration of Helsinki. All participants were provided with an information sheet and gave informed consent electronically before beginning the questionnaire.

### 2.3 Data collection and analysis

Data were gathered using an anonymous electronic questionnaire containing both open-ended and closed-ended questions, designed to collect both qualitative and quantitative information The full questionnaire in Czech language is provided in the Supplementary Appendix A. For reference purposes, an English translation of the questionnaire that was not used for data collection is provided in Supplementary Appendix B. The questionnaire was distributed online, and respondents could complete it voluntarily and anonymously over several months.

Quantitative data were analyzed using JASP (JASP Team, 2025) and Microsoft Excel. Analyses comprised exploratory data analysis (see Supplementary Appendix C) and descriptive statistical procedures to summarize the data and examine patterns, relationships, and distributions of the measured variables. Given the nature of the study and the limited sample size, any inferential statistical tests reported are interpreted as exploratory and hypothesis-generating rather than confirmatory. This analytical strategy enabled a systematic overview of participants’ responses and the identification of salient trends and associations in attitudes toward VR.

Qualitative data were analyzed using conventional qualitative content analysis following the principles described by Hsieh and Shannon (2005). First, all participant responses were read repeatedly to achieve immersion in the data and to gain a sense of the whole. Meaning units were then identified and labeled with initial codes. The codes were derived inductively from the data rather than from pre-existing theoretical categories. In the next step, conceptually similar codes were compared and grouped into subcategories and broader categories through an iterative process of constant refinement. Category labels and definitions were revised repeatedly to ensure that they accurately reflected participants’ accounts and remained grounded in the original data. The final analytic structure was developed through repeated review by the authors, with representative excerpts retained to illustrate each category.

## 3 Results

### 3.1 Key descriptives

**Figure 1:**
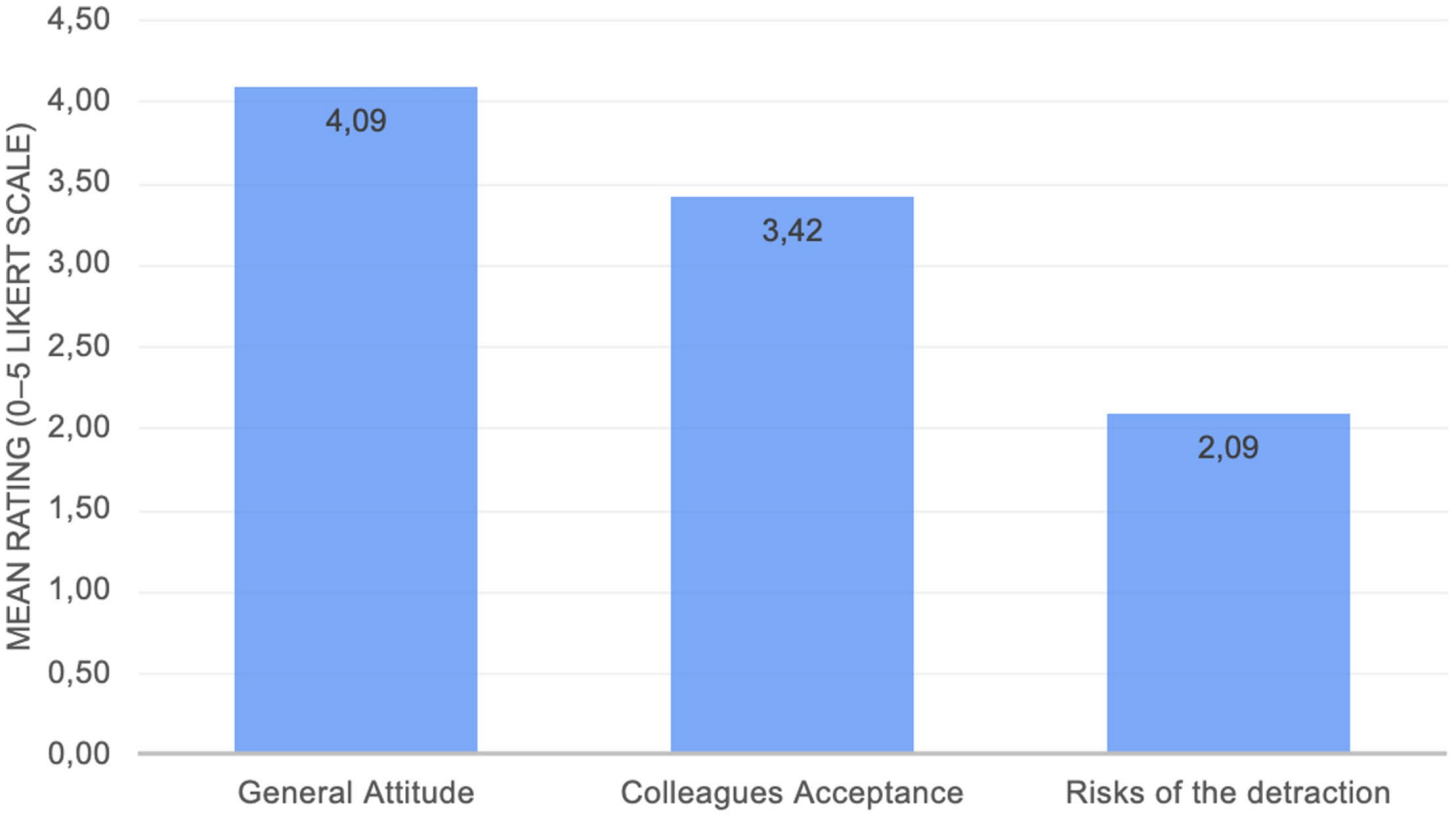
Overall results from the core inquired beliefs (scale ranging from 1 to 5).

Participants evaluated three core belief domains related to the use of VR in psychotherapeutic practice: (1) general attitude toward the use of VR in therapy, (2) perceived acceptance of VR among professional colleagues, and (3) perceived risks of potential detriment to the psychotherapeutic alliance associated with VR use. All constructs were assessed using items rated on a five-point Likert-type scale (ranging from 1 to 5). Descriptive statistics for each belief domain are presented in Figure 1.

#### 3.1.1 Overall attitude toward VR

Overall, therapists’ attitudes toward VR in psychotherapy were positive within the studied research sample. On a scale from 1 (“very negative”) to 5 (“very positive”), no respondent rated below 3, and the mean general attitude was approximately 4.1 (M = 4.09; SD = 0.72). Nearly half (48.5%) described their attitude as 4 (“fairly positive”), and about one-third (30.3%) chose 5 (“very positive”), with the remainder neutral (21.2%) rated 3.

#### 3.1.2 Perceived acceptance of VR among colleagues

Therapists were also asked to rate the current acceptance of VR among their colleagues on a 5-point scale (1 “not accepted at all” to 5 “widely accepted”). The perceived acceptance was moderate, centered around “somewhat accepted” (mode = 3). The average rating indicated, in general, that VR is seen as only partly accepted in their professional circles (M = 3.39; SD = 0.80). Only 18% of therapists perceived VR as highly accepted (ratings 4–5), whereas 9% considered its acceptance to be low (ratings 1–2).

#### 3.1.3 Perceived risk of detracting from the therapeutic alliance

In terms of potential downsides, therapists on average perceived only a low-to-moderate risk that VR might detract from the therapeutic alliance or other important aspects of therapy (M = 2.09, SD = 0.84) on a 5-point scale, where 5 = “very much” distraction risk. A majority (78.8%) rated this risk as 3 or below, suggesting that most do not feel VR would severely undermine the therapy relationship.

### 3.2 Perceived benefits and barriers

Regarding the potential benefits and barriers of VRET, participants were asked to select, from a predefined list of categories, which benefits they perceived virtual reality to offer over traditional exposure approaches in the treatment of acrophobia, including (1) greater environmental control, (2) safe exposure without the need for real-world situations, (3) gradual and flexible exposure tailored to the client’s pace, (4) better accessibility for clients with mobility or logistical constraints, (5) a stronger sense of safety, (6) lower costs and organizational demands, (7) repeated exposure under the same conditions, (8) higher client engagement, and (9) gamification. The most frequently endorsed benefit was an increased sense of safety for clients, reported by 28 participants (84.8%). This was followed by better accessibility for clients with mobility or logistical constraints (23 responses; 69.7%). Moderate levels of endorsement were observed for safe exposure without the need for real-world situations (21 responses; 63.6%) and greater environmental control (20 responses; 60.6%). More than half of the therapists also emphasized the possibility of tailoring exposure to the client’s pace and conducting repeated exposure under the same conditions (both 18 responses; 54.5%). A less commonly endorsed, though still notable, advantage was the potential reduction of costs and organizational demands compared with *in vivo* exposure (10 responses; 30.3%). The least frequently selected benefits were higher client engagement (9 responses; 27.3%) and gamification (1 response; 3.0%). Notably, no participant selected the option “I see no benefits,” indicating that every respondent identified at least one positive aspect of VR use in therapy.

Regarding the perceived barriers to the routine implementation of VRET, participants were asked to select, from a predefined list of categories, which obstacles they considered most important, including (1) high financial costs, (2) lack of specialized training, (3) technical complexity of operating VR equipment, (4) concerns about technical malfunctions during therapy, (5) uncertainty regarding the research evidence or long-term effects of VR, (6) limited availability of high-quality, validated applications in Czech/Slovak, (7) low compatibility with some clients, (8) preparation time, (9) concerns about a reduced quality of the therapeutic alliance, and (10) low client trust. Among these barriers, high financial costs emerged as the most frequently endorsed factor, reported by 20 participants (60.6%). This was followed by a cluster of technical, educational, and implementation-related concerns, each endorsed by 13 participants (39.4%), including lack of specialized training, technical complexity of operating VR equipment, concerns about technical malfunctions during therapy, limited availability of high-quality validated applications in Czech/Slovak, and low compatibility with some clients. Uncertainty regarding the research evidence or long-term effects of VR was also relatively common, reported by 10 participants (30.3%). Less frequently mentioned barriers included preparation time (4 responses; 12.1%), concerns about a reduced quality of the therapeutic alliance (3 responses; 9.1%), and low client trust (2 responses; 6.1%). Notably, only one participant (3.0%) indicated that they saw no barriers to the use of VR in therapy.

### 3.3 Additional findings

Additional noteworthy findings identified through inductive analysis of qualitative responses to the open-ended questions in the questionnaire are summarized in Table 1.

**Table 1:** Notifiable trends identified in the qualitative data.

Areas of improvement	Key findings
Clients refusing technology	Six therapists (18%) had encountered clients reluctant to engage with technology, while the majority (79%) reported that this was generally not the case.
Systemic support in the Czech Republic and Slovakia	Only 3 respondents (9%) perceived current systemic support as adequate; 17 (52%) were uncertain, and 13 (39%) considered it insufficient.
Key functional features for acrophobia applications	The most frequently mentioned desired features included: A *“stop button”* accessible to both client and therapist (82%), *Continuous height adjustment* (67%), *Automatic post-exposure reports* summarizing height, SUDS, and exposure duration (48%), And *a shared VR environment* allowing therapist–client co-presence (46%).
Shared environment (avatar functionality)	The perceived importance of a shared environment was moderate, with most responses clustered around “neutral” (score 3; 10 responses) and “less important” (score 2; 10 responses). Only two therapists rated this feature as essential.
Biofeedback integration	A substantial majority (79%) expressed interest in real-time biofeedback, particularly live heart rate monitoring, while a small minority (12%) were uninterested.
Need for VR session recordings	More than half of participants (57%) rated the usefulness of session recordings at 3–4 on a five-point scale, emphasizing their value for supervision and progress tracking.
Preferred training formats	Online training courses featuring practical demonstrations were the most preferred format (82%), followed by in-person workshops (45%) and supervision groups (27%).
Step-by-step methodological protocols	The majority of respondents (88%) expressed a strong interest in structured step-by-step methodological protocols, highlighting a clear need for standardized guidance in implementing VR-based therapy.

### 3.4 Qualitative findings

Inductive content analysis of participants’ written responses to the open-ended survey items identified three overarching themes: (1) infrastructure and training requirements, (2) scenario personalization and flexibility, and (3) ethical and technical concerns.

#### 3.4.1 Infrastructure and training requirements

Participants’ responses suggested that the implementation of VR in psychotherapy is not viewed only as a matter of clinician interest, but also as dependent on broader practical conditions, including access to suitable equipment, clarity regarding what tools should be used, and the availability of structured training. One participant explicitly highlighted the practical uncertainty surrounding implementation: “*I work online, so it is not practical. If I worked in person, I would not know what tools I should get for VR or where*” (Participant 16, translated from Czech/Slovak). Another respondent pointed to the ambiguity surrounding formal training and certification: “*Certified by whom? For example, in the Czech Republic, there is no certification for short courses. Should it exist? Yes*.” (Participant 21, translated from Czech/Slovak).

#### 3.4.2 Scenario personalization and flexibility

A second major theme concerned the need for VR applications to allow flexible adjustment of exposure scenarios to individual client needs. Participants emphasized control over the intensity, realism, and content of the virtual environment as key for therapeutic usefulness. For example, one participant described motivation to use VR in terms of “*greater control over the environment and the possibility to adjust the level of burden*” (Participant 18, translated from Czech/Slovak), while another emphasized “safety of the environment and the possibility to conduct exposure within the therapy setting” (Participant 10, translated from Czech/Slovak).

When asked about essential application features, participants also stressed the need for modifiable and realistic scenarios, for example: “*realistic conditions, presence of an avatar, graded levels of exposure*” (Participant 14, translated from Czech/Slovak), and “*modulation of factors, affordability, safety of the environment*” (Participant 17, translated from Czech/Slovak). One participant additionally suggested that therapeutic value could be increased by enabling “*exposure according to the client’s own imagination*” (Participant 8, translated from Czech/Slovak). Taken together, these responses indicate that therapists do not view VR merely as a static exposure tool, but as an intervention platform that should support individualized and dynamically adjustable treatment.

#### 3.4.3 Ethical and technical concerns

The third theme captured participants’ concerns about the reliability, realism, affordability, and clinical transferability of VR-based interventions. Several respondents questioned whether VR exposure would generalize well to real-life situations. For instance, one participant noted: “*The only thing is that the client may then fail in the real situation*” (Participant 5, translated from Czech/Slovak), while another stated: “*That it would not be as effective as in vivo exposure*” (Participant 8, translated from Czech/Slovak). Concerns about realism were also evident in responses such as “*VR environments still look very unrealistic; I am not sure that this would help prepare for reality*” (Participant 18, translated from Czech/Slovak) and “*cost, insufficient realism*” (Participant 10, translated from Czech/Slovak).

Technical reliability and cost were likewise recurring issues, including brief but clear references to “*problems with unstable signal*” (Participant 7, translated from Czech/Slovak), “*quality of the technology*” (Participant 11, translated from Czech/Slovak), and “*higher acquisition costs*” (Participant 17, translated from Czech/Slovak). Overall, these findings suggest that participants’ reservations were not limited to technology itself, but also concerned whether VR can provide a clinically credible, safe, and transferable therapeutic experience.

## 4 Discussion

This study provides an initial, context-specific snapshot of CBT psychotherapists’ readiness to engage with VR in the Czech Republic and Slovakia. Consistent with the descriptive results, VRET remains largely unfamiliar in practice, with only 15% reporting any prior therapeutic VR use, but respondents expressed consistently positive attitudes toward VR as a therapeutic tool: none rated their overall attitude below 3 on a 5-point scale, and the mean rating was 4.09. This pattern may suggest a “readiness-implementation gap”: clinicians may endorse VR conceptually while lacking the conditions to integrate it safely and efficiently into everyday care. A related finding is that perceived acceptance of VR among colleagues was only moderate, which may indicate that, despite generally favorable personal attitudes, respondents still perceive VR as not yet fully normalized within their broader professional communities. This pattern is in line with the still limited routine uptake of VRET and may reflect continuing unfamiliarity with its clinical use. Descriptive differences by gender were small across all three core belief variables (Table 2).

**Table 2:** Group descriptives for gender differences.

Variable	Group	*N*	Mean	SD	SE	Coefficient of variation
General attitude	Female	24	4.083	0.776	0.158	0.190
	Male	9	4.111	0.601	0.200	0.146
Colleagues attitude	Female	24	3.292	0.859	0.175	0.261
	Male	9	3.667	0.500	0.167	0.136
Risks of detraction	Female	24	2.208	0.833	0.170	0.377
	Male	9	1.778	0.833	0.278	0.469

Perceived benefits from quantitative part of survey clustered around therapeutic controllability and client safety. A clear majority of participants emphasized VR’s capacity to enhance client safety and accessibility, and many highlighted the ability to precisely calibrate exposure intensity, repeat scenarios under consistent and well-controlled conditions and reduce the logistical burden of in-vivo exposure, advantages that align with proposed VRET mechanisms (e.g., habituation, expectancy violation, and gains in self-efficacy). By contrast, very few respondents viewed “gamification” or engagement-oriented features as important. This may suggest that clinicians in this context prioritize usability, controllability, and therapeutic relevance more strongly than features intended to increase user engagement. Differences by previous VR experience were also small, with slightly more favorable ratings among respondents with prior VR experience (Table 3).

**Table 3:** Group descriptives for previous VR experience.

Variable	Group	*N*	Mean	SD	SE	Coefficient of variation
General attitude	Yes	5	4.400	0.548	0.245	0.124
	No	28	4.036	0.744	0.141	0.184
Colleagues attitude	Yes	5	3.600	0.894	0.400	0.248
	No	28	3.357	0.780	0.147	0.232
Risks of detraction	Yes	5	2.200	0.837	0.374	0.380
	No	28	2.071	0.858	0.162	0.414

Barriers in closed questions were dominated by financial cost and closely followed by training, technical demands, and limited access to appropriate therapeutic content. The prominence of cost concerns, reinforced by the majority who reported that financial considerations shape adoption decisions, underscores that willingness alone may be insufficient without reimbursement pathways, institutional investment, or lower-cost procurement models, as participants stated. In the quantitative part, a high proportion of participants reported wanting step-by-step methodological protocols and expressed a preference for online training with practical demonstrations. On this basis, we hypothesize that scalable, practice-oriented implementation packages incorporating clinical decision rules, safety procedures, contraindication screening, and troubleshooting alongside exposure planning could be particularly valuable in supporting the adoption of VR in psychotherapy. Open-ended responses also identified limited scenario personalization and flexibility as a barrier, with participants emphasizing the need for modifiable, realistic, and individually tailored VR environments. In addition, therapists raised ethical and technical concerns related to realism and transferability to real-life situations, suggesting that reservations about VR could be linked not only to the technology itself but also to its perceived clinical credibility and usefulness.

An informative nuance emerged in perceived risk of VR detracting from the therapeutic alliance. Overall, respondents tended to perceive this risk as relatively low, suggesting that most did not expect VR to substantially undermine the therapeutic relationship. This interpretation is encouraging, as it indicates that concerns about disruption of rapport may not represent a major barrier to adoption in this sample, although some degree of caution clearly remains. Although overall risk ratings were low to moderate, the observed relationship with practice length followed a U-shaped pattern, with mid-career therapists reporting the lowest perceived risk and both novice and very experienced clinicians expressing greater caution. Because this relationship emerged from post-hoc analyses of a small, convenience-based sample and did not reach statistical significance, it should be regarded as exploratory. Replication with larger and more balanced samples is necessary before drawing firm conclusions about how professional experience moderates perceptions of VR-related risks.

In the closed-ended items, respondents’ feature preferences provide concrete design requirements for acrophobia-focused applications and likely generalize to other exposure scenarios. A universally accessible “stop button,” smooth intensity control (height adjustment), automated post-session summaries, and optional biofeedback align with a clinician’s need for safety, pacing, and documentation. Interest in biofeedback also may suggest a pathway to strengthen case formulation and collaborative empiricism by making physiological arousal visible and trackable.

Several limitations should be considered. The sample was small, convenience-based, and skewed toward early-career clinicians, limiting generalizability and statistical power; inferential tests should therefore be treated as hypothesis-generating. All data were self-reported and cross-sectional, which may be influenced by selection effects (e.g., therapists interested in innovation may have been more likely to respond) and cannot establish causal relationships (e.g., whether VR experience increases positivity or whether positive clinicians seek VR experience). The “>10 years” subgroup was very small, so the practice-length effects require replication with balanced samples. Future research would benefit from larger, more representative sampling, validated attitude and implementation-readiness measures, and longitudinal designs that assess changes before and after hands-on VR workshops or pilot implementations in clinics. Mixed-methods implementation trials, including economic evaluation and workflow impact, would further clarify the conditions under which VR moves from interest to sustained adoption.

In real-world clinical settings, the proposed VR intervention could be implemented as a structured adjunct to standard CBT, particularly in services already delivering exposure-based treatment. Its practical use would likely require brief therapist training, clear step-by-step protocols, reliable technical support, and affordable hardware and software. Initial implementation may be most feasible with standardized applications for specific phobias, with later adaptation to more individualized scenarios as clinician experience and institutional support increase.

## 5 Conclusion

CBT psychotherapists in the Czech Republic and Slovakia appear open to VR and largely do not anticipate major harm to the therapeutic alliance, yet VR remains rarely used in practice. The main obstacles are structural, especially cost, access to training, and technical and organizational feasibility, rather than ideological resistance. Clinicians also expressed a clear need for standardized, step-by-step methodological guidance and practical training formats, alongside tools designed around safety, controllability, documentation, and optional psychophysiological monitoring.

Taken together, the findings suggest that accelerating VRET uptake in this region will depend on implementation ecosystems: affordable access to hardware/software, reimbursement or institutional support, clinician-centered training and supervision pathways, and co-designed VR applications that fit CBT workflows. Pilot programs that provide supervised hands-on experience, paired with pragmatic protocols and reliable technical support, may be a high-leverage route to convert positive attitudes into routine clinical use.

## Data Availability

The data that support the findings of this study are available from the corresponding author upon reasonable request. Due to ethical and privacy considerations, the data are not publicly available.
