# Supplementary material for: CBT therapists’ attitudes toward virtual reality use in psychotherapy: a brief report from the Czech Republic and Slovakia
Source: Front Psychol. 2026 May 7;17:1811278. doi: 10.3389/fpsyg.2026.1811278 (PMC13190445; doi:10.3389/fpsyg.2026.1811278)
Supplement: Supplementary file 1 [file Data_Sheet_1.pdf]

Postoje a potreby KBT terapeutov z Česka a Slovenska k implementácii virtuálnej reality (VR) do terapeutickej praxe

Vážená pani terapeutka, vážený pán terapeut,

dovoľte nám úvodom poďakovať za Váš čas a ochotu zúčastniť sa nášho prieskumu zameraného na postoje a potreby kognitívno-behaviorálnych (KBT) terapeutov z Česka a Slovenska, ktorí sa aktívne venujú individuálnej KBT terapii.

Virtuálna realita (VR) je technológia, ktorá umožňuje používateľovi ponoriť sa do počítačom vytvoreného trojrozmerného prostredia. V kontexte expozičnej terapie je možné pomocou VR zobrazovať realistické vizualizácie obávaných situácií v bezpečnom a kontrolovanom prostredí. Takto možno nahradiť alebo doplniť imagináciu a zároveň ušetriť čas a námahu spojenú so zháňaním reálnych stimulov.

Cieľom prieskumu je:

- **zmapovať váš postoj** k využívaniu VR pri terapeutickej expozícii (napríklad pri liečbe akrofóbie a iných fóbií),

- zistiť, aké bariéry môžu brániť zavedeniu VR do praxe,

- **identifikovať potreby** a požiadavky na vzdelávanie, technickú či metodickú podporu,

- a zároveň **odhaľovať** kľúčové vlastnosti, ktoré by mala VR aplikácia spĺňať, aby bola pre terapeutov užitočná.

Dotazník je anonymný a jeho vyplnenie trvá približne **10–15 minút.** Vaše odpovede poslúžia na vedecké účely, budú spracované a prezentované len v súhrnnej podobe. Výsledky prieskumu nám pomôžu lepšie pochopiť, ako vyjsť v ústrety terapeutom, ktorí chcú (alebo zvažujú) využiť možnosti VR pri svojej každodennej práci s klientmi.

Vopred vám ďakujeme za váš prínos a úprimné odpovede. V prípade akýchkoľvek otázok nás môžete kontaktovať na kristinavars@gmail.com.

S úctou,

Kristina Kvapil Varšová

* Označuje povinnú otázku

1. Vaše pohlavie *

Označte iba jednu elipsu.

- Žena
- Muž
- Iné: ___

2. Dĺžka psychoterapeutickej praxe *

Označte iba jednu elipsu.

- Menej než 5 rokov
- 5-10 rokov
- Viac ako 10 rokov

3. Máte už osobnú skúsenosť s využívaním VR v terapii (napr. pri inom probléme)? *

Označte iba jednu elipsu.

- Áno
- Nie

Celkový postoj k integrácii VR technológií do klinickej praxe

4. Ako by ste opísali váš všeobecný postoj k využívaniu virtuálnej reality v psychoterapii? *

Označte iba jednu elipsu.

	1	2	3	4	5	
Úplr	◯	◯	◯	◯	◯	Silné presvedčenie o prínose VR

5. Aké potenciálne prínosy vnímate pri použití VR oproti tradičným prístupom expozície (napr. pri akrofóbii)? *

Začiarknite všetky vyhovujúce možnosti.

- [ ] Lepšia kontrola prostredia
- [ ] Vyššie zapojenie klienta
- [ ] Možnosť bezpečnej expozície bez nutnosti reálnych výjazdov do výšok
- [ ] Možnosť postupnej a flexibilnej expozície podľa tempa klienta
- [ ] Lepšia dostupnosť pre klientov s obmedzenou mobilitou alebo logistikou
- [ ] Zvýšený pocit bezpečia pre klienta vďaka kontrolovanému prostrediu
- [ ] Zníženie nákladov a organizačnej náročnosti expozície v reálnom prostredí
- [ ] Možnosť opakovanej expozície v rovnakých podmienkach
- [ ] Nevnímam žiadne možné pozitíva
- [ ] Iné: ____________________

6. Čo vás najviac motivuje (alebo by motivovalo) k vyskúšaniu VR v terapeutickej praxi?

____
____
____
____
____

7. Sú nejaké konkrétne obavy alebo neistoty, ktoré u vás vyvoláva predstava využívania VR počas terapie?

____
____
____
____
____

8. Ak by ste mali vyjadriť, do akej miery je VR v súčasnosti akceptovaná medzi vašimi kolegami, ako by ste to zhodnotili? *

Označte iba jednu elipsu.

	1	2	3	4	5	
Vôb	◯	◯	◯	◯	◯	Väčšina to víta

Vnímané bariéry a obmedzenia pri prijatí VR do terapie

9. Aké hlavné bariéry vidíte v tom, aby sa VR technológie stali bežnou súčasťou kognitívno-behaviorálnej terapie? *

Začiarknite všetky vyhovujúce možnosti.

- [ ] Vysoké finančné náklady
- [ ] Nedostatok špecializovaného výcviku
- [ ] Nízka dôvera klientov
- [ ] Čas na prípravu
- [ ] Technická náročnosť obsluhy VR zariadení
- [ ] Obavy z technických porúch počas terapie
- [ ] Obava zo zníženia kvality terapeutickej aliancie
- [ ] Neistota ohľadom výskumnej opory alebo dlhodobých efektov VR
- [ ] Nízka dostupnosť kvalitných, overených aplikácií v češtine/slovenčine
- [ ] Nízka kompatibilita s niektorými klientmi (napr. vek, diagnóza, technická gramotnosť)
- [ ] Nevidím bariéry
- [ ] Iné: ____________________

10. Myslíte si, že finančné hľadisko (cena hardvéru, softvéru) môže zásadne ovplyvniť rozhodnutie terapeutov o integrácii VR? *

Označte iba jednu elipsu.

- Áno
- Nie
- Neviem
- Iné:

11. Do akej miery vnímate riziko, že VR by mohla odvádzať pozornosť od terapeutickej aliancie alebo iných dôležitých aspektov terapie? *

Označte iba jednu elipsu.

	1	2	3	4	5	
Vôb						Veľmi výrazne

12. Aké technické komplikácie (napr. nevoľnosť klienta, nestabilita softvéru, problémy s pripojením) považujete za najkritickejšie?

13. Stretli ste sa už v praxi s klientmi, ktorí vyslovene odmietajú technológie? *

Označte iba jednu elipsu.

- Áno
- Nie
- Iné:

14. Je podľa vás v Česku alebo na Slovensku dostatočná podpora pre implementáciu VR do psychoterapie (napr. cez odborné spoločnosti, výskumné projekty, granty)? *

Označte iba jednu elipsu.

- Áno
- Nie
- Neviem
- Iné:

Kľúčové vlastnosti a funkcie VR aplikácie

15. Ktoré funkcie by ste považovali za absolútny základ pre efektívne využitie VR v expozícii pri akrofóbii? *

Začiarknite všetky vyhovujúce možnosti.

- [ ] Riadenie úrovne výšky
- [ ] Záznam priebehu expozície
- [ ] Možnosť monitorovať fyziologické reakcie
- [ ] Možnosť spoločného pobytu terapeuta a klienta vo VR prostredí (napr. formou avatarov)
- [ ] Stop tlačidlo pre klienta aj terapeuta
- [ ] Možnosť nastavovať intenzitu prostredia (napr. vietor, sklon, pohyb podlahy, zvuk)
- [ ] Vizualizácia priebežného stresu/úzkosti (napr. SUDS v prostredí)
- [ ] Automatické generovanie reportu po expozícii (napr. dosiahnutá výška, úroveň úzkosti, trvanie)
- [ ] Možnosť integrácie relaxačného režimu po expozícii (napr. guided meditation, dýchacie cvičenia)
- [ ] Viacjazyčná podpora
- [ ] Iné: ______________________

16. Aká dôležitá je pre vás možnosť, aby sa terapeut s klientom nachádzali v spoločnom virtuálnom prostredí (vo forme avatarov)? *

Označte iba jednu elipsu.

	1	2	3	4	5	
Vôb	◯	◯	◯	◯	◯	Kľúčové

17. Do akej miery považujete za dôležité mať v aplikácii možnosť pre terapeutov „zasiahnuť" do virtuálneho prostredia (napr. pridať rôzne podnety alebo meniť rýchlosť výťahu)? *

Označte iba jednu elipsu.

	1	2	3	4	5	
Vôb	◯	◯	◯	◯	◯	Je to veľmi dôležité

18. Chceli by ste v aplikácii merať a zobrazovať fyziologické údaje klienta (napr. srdcový tep) v reálnom čase? *

Označte iba jednu elipsu.

- Áno
- Nie
- Iné:

19. Do akej miery je pre vašu prácu potrebný záznam z VR (videozáznam, prípadne záznam fyziologických reakcií) na neskoršie vyhodnotenie či supervíziu? *

Označte iba jednu elipsu.

	1	2	3	4	5	
Vôb						Veľmi potrebný

20. Máte nápad na ďalšie funkcie, ktoré by mohli zvýšiť terapeutickú hodnotu VR aplikácie (napr. systém odmien, gamifikácia, sprievodné audio-inštrukcie a pod.)?

21. Ak by ste mali určiť TOP 3 nevyhnutné vlastnosti VR aplikácie pre úspešnú terapiu akrofóbie, ktoré by to boli?

Potreba vzdelávania a odbornej podpory

22. Aká forma vzdelávania alebo tréningu by bola pre vás najatraktívnejšia, aby ste začali alebo zlepšili svoje schopnosti pracovať s VR? *

Začiarknite všetky vyhovujúce možnosti.

- [ ] Workshopy naživo
- [ ] Online kurzy s praktickými ukážkami
- [ ] Supervízne skupiny
- [ ] Čítanie prípadových štúdií
- [ ] Iné: ____________________

23. Myslíte si, že by malo existovať oficiálne certifikované školenie pre terapeutov, ktorí chcú využívať VR? *

Označte iba jednu elipsu.

- ( ) Áno
- ( ) Nie
- ( ) Iné: ____

24. Do akej miery by ste ocenili prítomnosť metodických materiálov či protokolov (napr. „Ako krok za krokom aplikovať VR pre akrofóbiu“)? *

Označte iba jednu elipsu.

	1	2	3	4	5	
Vôb	( )	( )	( )	( )	( )	Je to zásadné

Tento obsah nie je vytvorený ani schválený spoločnosťou Google.

Google Formuláre
