# Supplementary material for: CBT therapists’ attitudes toward virtual reality use in psychotherapy: a brief report from the Czech Republic and Slovakia
Source: Front Psychol. 2026 May 7;17:1811278. doi: 10.3389/fpsyg.2026.1811278 (PMC13190445; doi:10.3389/fpsyg.2026.1811278)
Supplement: Supplementary file 2 [file Data_Sheet_2.pdf]

Supplementary Appendix B: Attitudes and Needs of CBT Therapists in the Czech Republic and Slovakia Regarding the Implementation of Virtual Reality (VR) in Therapeutic Practice

English translation of the full questionnaire – not used in data collection

Dear Therapist,

First, we would like to thank you for your time and willingness to participate in our survey focused on the attitudes and needs of cognitive-behavioral therapy (CBT) therapists from the Czech Republic and Slovakia who actively provide individual CBT therapy.

Virtual reality (VR) is a technology that allows the user to immerse themselves in a computer-generated three-dimensional environment. In the context of exposure therapy, VR can be used to present realistic visualizations of feared situations in a safe and controlled setting. In this way, it can replace or complement imagination-based exposure while saving the time and effort associated with obtaining real-life stimuli.

The aim of the survey is to:

- map your attitude toward the use of VR in therapeutic exposure (for example, in the treatment of acrophobia and other phobias),
- identify barriers that may hinder the introduction of VR into practice,
- identify needs and requirements for training, technical support, and methodological support,
- and identify the key features that a VR application should have in order to be useful for therapists.

The questionnaire is anonymous, and completing it takes approximately 10-15 minutes. Your responses will be used for scientific purposes and will be processed and presented only in aggregate form.

The results of the survey will help us better understand how to meet the needs of therapists who want to use, or are considering using, the possibilities of VR in their everyday work with clients.

Thank you in advance for your contribution and honest answers. If you have any questions, you may contact us at kristinavars@gmail.com.

Sincerely,
Kristina Kvapil Varsova

1. Your gender *

Select only one option.

□ Female

□ Male

□ Other:

2. Length of psychotherapeutic practice *

Select only one option.

□ Less than 5 years

□ 5-10 years

□ More than 10 years

3. Do you already have personal experience using VR in therapy (e.g., for another problem)? *

Select only one option.

□ Yes

□ No

Overall attitude toward integrating VR technologies into clinical practice

4. How would you describe your general attitude toward the use of virtual reality in psychotherapy? *

Complete rejection	1	2	3	4	5	Strong belief in the benefits of VR
	○	○	○	○	○	

5. What potential benefits do you perceive in using VR compared with traditional exposure approaches (e.g., in acrophobia)? *

Select all that apply.

□ Better control of the environment

□ Greater client engagement

□ Possibility of safe exposure without the need for real trips to heights

□ Possibility of gradual and flexible exposure according to the client's pace

□ Better accessibility for clients with limited mobility or logistical constraints

□ Increased sense of safety for the client due to the controlled environment

□ Reduced costs and organizational demands of exposure in a real environment

□ Possibility of repeated exposure under the same conditions

□ I do not perceive any potential benefits

□ Other:

6. What motivates you most, or what would motivate you, to try VR in therapeutic practice?

7. Are there any specific concerns or uncertainties that the idea of using VR during therapy raises for you?

Open question.

8. If you were to express the extent to which VR is currently accepted among your colleagues, how would you rate it? *

Select only one option.

Not at all	1	2	3	4	5	Most welcome it

	○	○	○	○	○	

Perceived barriers and limitations in adopting VR in therapy

9. What main barriers do you see to VR technologies becoming a common part of cognitive-behavioral therapy? *

Select all that apply.

□ High financial costs

□ Lack of specialized training

□ Low client trust

□ Time needed for preparation

□ Technical difficulty of operating VR devices

□ Concerns about technical failures during therapy

□ Concern about reduced quality of the therapeutic alliance

□ Uncertainty regarding research support or the long-term effects of VR

□ Low availability of high-quality, validated applications in Czech/Slovak

□ Low compatibility with some clients (e.g., age, diagnosis, technological literacy)

□ I do not see any barriers

□ Other:

10. Do you think the financial aspect (cost of hardware and software) can fundamentally influence therapists' decisions about integrating VR? *

Select only one option.

□ Yes

□ No

□ I do not know

□ Other:

11. To what extent do you perceive a risk that VR could distract from the therapeutic alliance or other important aspects of therapy? *

Select only one option.

Not at all	1	2	3	4	5	Very strongly
	○	○	○	○	○	

12. Which technical complications (e.g., client nausea, software instability, connectivity problems) do you consider the most critical?

Open question.

13. Have you already encountered clients in practice who explicitly refuse technology? *

Select only one option.

□ Yes

□ No

□ Other:

14. Do you think there is sufficient support in the Czech Republic or Slovakia for the implementation of VR in psychotherapy (e.g., through professional societies, research projects, grants)? *

Select only one option.

□ Yes

□ No

□ I do not know

□ Other:

Key properties and features of a VR application

15. Which features would you consider the absolute foundation for the effective use of VR in exposure therapy for acrophobia? *

Select all that apply.

□ Control of height level

□ Recording of the exposure session

□ Ability to monitor physiological responses

□ Possibility for therapist and client to be present together in the VR environment (e.g., in the form of avatars)

□ Stop button for both client and therapist

□ Ability to adjust environmental intensity (e.g., wind, incline, floor movement, sound)

□ Visualization of ongoing stress/anxiety (e.g., SUDS in the environment)

□ Automatic generation of a report after exposure (e.g., height reached, level of anxiety, duration)

□ Possibility of integrating a relaxation mode after exposure (e.g., guided meditation, breathing exercises)

□ Multilingual support

□ Other:

16. How important is it for you that the therapist and client can be present in a shared virtual environment (in the form of avatars)? *

Select only one option.

Not at all	1	2	3	4	5	Essential
	○	○	○	○	○	

17. To what extent do you consider it important for therapists to have the ability to "intervene" in the virtual environment (e.g., add different stimuli or change the speed of the elevator)? *

Select only one option.

Not important at all	1	2	3	4	5	Very important
	○	○	○	○	○	

18. Would you like the application to measure and display the client's physiological data (e.g., heart rate) in real time? *

Select only one option.

□ Yes

□ No

□ Other:

19. To what extent is a recording from VR (video recording and/or recording of physiological responses) needed in your work for later evaluation or supervision? *

Select only one option.

Not at all	1	2	3	4	5	Very necessary
	○	○	○	○	○	

20. Do you have ideas for additional features that could increase the therapeutic value of a VR application (e.g., reward system, gamification, accompanying audio instructions, etc.)?

Open question.

21. If you had to specify the TOP 3 essential features of a VR application for successful therapy of acrophobia, what would they be?

Open question.

Need for training and professional support

22. What form of education or training would be most attractive to you in order to start using VR or improve your ability to work with it? *

Select all that apply.

□ In-person workshops

□ Online courses with practical demonstrations

□ Supervision groups

□ Reading case studies

□ Other:

23. Do you think there should be official certified training for therapists who want to use VR? *

Select only one option.

□ Yes

□ No

□ Other:

24. To what extent would you appreciate the availability of methodological materials or protocols (e.g., "How to apply VR for acrophobia step by step")? *

Select only one option.

Not needed at all	1	2	3	4	5	Essential
	○	○	○	○	○	
