# Supplementary material for: CBT therapists’ attitudes toward virtual reality use in psychotherapy: a brief report from the Czech Republic and Slovakia
Source: Front Psychol. 2026 May 7;17:1811278. doi: 10.3389/fpsyg.2026.1811278 (PMC13190445; doi:10.3389/fpsyg.2026.1811278)
Supplement: Supplementary file 3 [file Data_Sheet_3.pdf]

Supplementary Appendix C: Group comparisons - Exploratory analysis

In order to inform future research directions, we conducted an exploratory analysis focusing on potential differences across a range of variables. A one-way between-groups analysis of variance (ANOVA) was conducted to examine differences in perceived risks of detraction as a function of psychotherapeutic practice length, categorized as *less than 5 years*, *5–10 years*, and *more than 10 years*. Descriptive statistics indicated a non-linear pattern across groups, with the lowest mean level of perceived risk reported by therapists with 5–10 years of practice (M = 1.40, SD = 0.70), compared to those with less than 5 years (M = 2.35, SD = 0.67) and those with more than 10 years of practice (M = 2.67, SD = 1.16). The assumption of homogeneity of variances was met, as indicated by Levene's test, $F(2, 30) = 1.24$, $p = .304$. The classical ANOVA revealed a statistically significant effect of practice length on perceived risks of detraction, $F(2, 30) = 6.83$, $p = .004$, with a large effect size ($\omega^2 = .26$), suggesting that approximately one quarter of the variance in perceived risk was attributable to differences in practice length. Given the markedly unequal group sizes - particularly the very small number of participants in the *more than 10 years* group ($n = 3$) - a robustness check was conducted using Welch's ANOVA, which does not assume equal variances or balanced designs. Welch's test likewise indicated a significant effect of practice length, $F(2, 5.06) = 5.95$, $p = .047$.

Post-hoc pairwise comparisons were examined using Tukey's HSD procedure. These comparisons indicated that therapists with less than 5 years of practice reported significantly higher perceived risks of detraction than those with 5–10 years of practice (mean difference = 0.95, p = .005). In addition, therapists with more than 10 years of practice reported significantly higher perceived risks than those in the 5–10 years group (mean difference = −1.27, p = .032). No significant difference was observed between therapists with less than 5 years and more than 10 years of practice (mean difference = −0.32, p = .760). This pattern corresponds to a U-shaped relationship, with the lowest perceived risk observed among mid-career therapists. See Figure 2. A one-way ANOVA did not reveal any effect of psychotherapeutic practice length on other core beliefs, such as general attitudes toward VR ($F(2, 30) = 0.237$, $p = .790$, $\omega^2 = 0.000$) or perceived colleagues' attitudes toward VR ($F(2, 30) = 1.689$, $p = .202$, $\omega^2 = 0.040$).

Regarding gender, men and women did not differ in their overall attitude scores (Welch's $t(18.59) = -0.109$, $p = .915$, Hedges' $g = -0.04$), indicating a negligible effect. Likewise, no between-group differences were observed for perceived colleagues' attitudes (Welch's $t(24.89) = -1.550$, $p = .134$, Hedges' $g = -0.52$), corresponding to a moderate effect size, or for perceived risks of detraction from the therapeutic alliance (Welch's $t(14.41) = 1.322$, $p = .207$, Hedges' $g = 0.50$), also reflecting a moderate effect. See the descriptives in Table 2.

With respect to previous experience with VR, those who have experienced VR before did not differ from those who haven't in their overall attitude scores (Welch's $t(6.962) = 1.290$, $p = .238$, Hedges' $g = 0.54$), indicating a moderate effect size despite the non-significant result. Likewise, no between-group differences were observed for perceived colleagues' attitudes (Welch's $t(5.146) = 0.570$, $p = .593$, Hedges' $g = 0.28$), corresponding to a small effect, or for perceived risks of detraction from the therapeutic alliance (Welch's $t(5.612) = 0.315$, $p = .764$, Hedges' $g = 0.15$), reflecting a negligible effect. See the descriptives in Table 3.

No significant difference in enthusiasm ($t(31)=1.20$, p = .24) was found between therapists who had personal VR experience (mean attitude 4.4) and those without VR experience (mean 4.0).

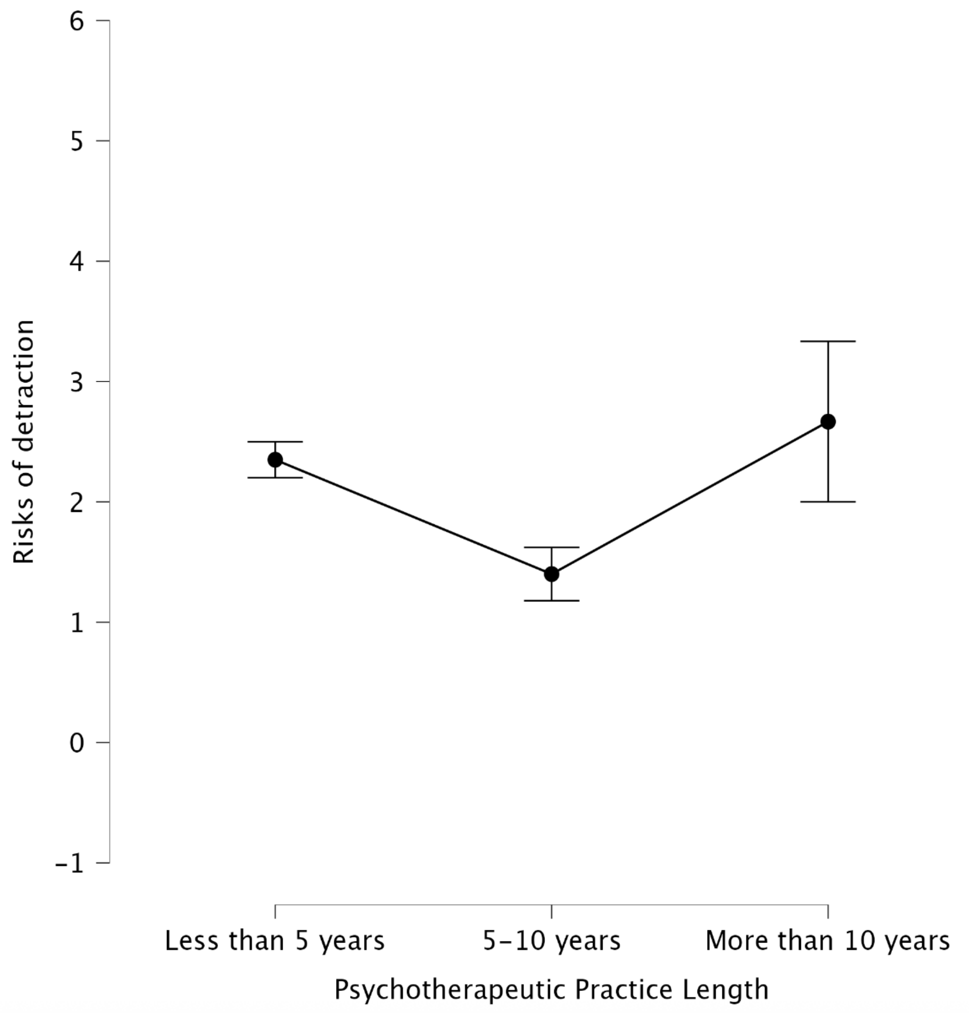

Figure 2. Perceived risk of detraction within therapeutic process with respect to the length of psychotherapeutic practice (error bars represent standard
